# Mothers’ utilization and associated factors in preconception care in northern Ethiopia: a community based cross sectional study

**DOI:** 10.1186/s12884-019-2478-1

**Published:** 2019-10-11

**Authors:** Tsrity Tadese Asresu, Desta Hailu, Berhe Girmay, Mulugeta Woldu Abrha, Haftom Gebrehiwot Weldearegay

**Affiliations:** 1 Mekelle University, College of Health Sciences, Mekelle, Ethiopia; 2 Mekelle University, College of Health Sciences, School of Nursing, Mekelle, Ethiopia; 3 Tigray Health Research Institute, Mekelle, Ethiopia; 4 Department of Midwifery, Mekelle University, College of Health Sciences, Mekelle, Ethiopia

**Keywords:** Preconception care, Adverse birth outcomes, Mekelle City, Ethiopia

## Abstract

**Background:**

Adverse pregnancy outcomes remain a prevalent health problem in Ethiopia. Mothers’ use of preconception care service has the potential to avert many of the adverse outcomes. However, the use of this service and its determinants is not well investigated. Therefore, this study was conducted to assess the utilization and determinants of preconception care among recently delivered mothers.

**Methods:**

A community based cross-sectional design was conducted among 564 recently delivered mothers in Mekelle City, Northern Ethiopia. A multi stage cluster sampling technique was employed. Data was collected using a pre-tested, structured interviewer questionnaire and was entered in to Epi-Info™ Version 7 and analyzed using SPSS™ Version 20.0. Descriptive, bivariable and multivariable logistic regression was used to identify the association.

**Results:**

This study revealed that, 102(18.2%) of the mothers had utilized preconception care. Mothers’ knowledge on preconception care (AOR: 2.21; 95% CI: 1.03, 4.73), prior experience of adverse birth outcomes (AOR: 5.10; 95% CI: 2.31, 11.24), history of chronic health problems (AOR: 5.69; 95% CI: 2.06, 15.72), husband’s support (AOR: 13.84; 95% CI: 6.02, 31.79), and challenges in accessing a health facility (AOR: 0.24; 95% CI: 0.16, 0.48) were significantly associated with preconception care service utilization.

**Conclusion:**

Mothers’ utilization of preconception care is low. Mothers knowledge on preconception care, experience of adverse birth outcome, having chronic health problems and husband support increases utilization of preconception care. However, mothers who experienced challenges in visiting a health facility showed decrease preconception care utilization. Therefore, increased efforts are need in terms of advocating for involvement of husband’s and awareness creation respecting preconception care services for all women.

## Background

Care given to a couple to ensure they are in good health before they conceive a child is an essential component to optimize the health and development of future offspring. Preconception care (PCC) improves the health of women and men while reducing the chances that their children will experience: prematurity, low birth weight, birth defects or other birth-related conditions that could hinder optimal child development [1, 2]. Preconception care is a critical component of health which includes the provision of biomedical, behavioral and social health interventions to women of reproductive age and couples before they conceive [3, 4].

The World Health Organization has recommended a package of interventions for PCC including maternal nutrition such as micronutrient supplementation (iron, folic acids and others), vaccination, cessation of tobacco and excessive alcohol use, prevention of interpersonal violence, sexuality education, protection from environmental hazards, genetic counseling, and support for mental health. Adolescence is a prime – though not the only – window of opportunity to deliver these interventions [4, 5].

This risk of maternal and infant mortality and pregnancy-related complications can be reduced by increasing access to quality preconception (before pregnancy) and interconception (between pregnancies) care [6]. Preconception care has a positive impact on reduction in mortality and decrease the risk of adverse health effects for the woman, fetus, and neonate by optimizing the woman’s health and knowledge before planning and conceiving a pregnancy [6, 7]. Moreover, Interventions before pregnancy can increase the health and well-being of adolescents, adult women and men, and improve subsequent pregnancy and child health outcomes [8, 9]. This evidence indicated that, PCC is one of the evidence-based health promotion interventions to prevent adverse pregnancy outcomes. Nevertheless, it is one of the missing elements within the continuum of maternal and child health care [10, 11].

Although the government of Ethiopia has been working to improve coverage and access to institutional delivery and postpartum care over the past couple of decades, adverse pregnancy outcomes, specifically neural tube related defects such as anencephaly, spinal bifida, and encephalocoele, are increasing at an alarming rate. Furthermore, PCC service is provided in both hospitals and health centers in Ethiopia. However, there are concerns about standardize and regular PCC service provided for all reproductive age group mothers [8, 12].

To date, a limited number of studies have considered on factors associated with the mother’s utilization of PCC service in Ethiopia. Therefore, this study sought to determine the prevalence of mothers’ utilization and associated factors of PCC and will contribute to the design of effective preventive strategies to tackle the rising burden of adverse pregnancy and birth outcomes.

## Methods

### Study setting and participants

A community based cross-sectional study design was conducted between March 15th and April 30th, 2018 in Mekelle City, which is the capital city of Tigray Regional State, Ethiopia. Mekelle city is divided into AddiHak’i, Ayder, Haddinet, Hawelti, Qedamay Weyyane, Kwiha, and Semien sub-cities and also subdivided into 33 Tabias.^1^ According to the 2017 report, a total of 412,938 people reside in Mekelle city with 23.48% percent being of the female reproductive age group (i.e., 15–49 years). The source of population were all delivered mothers in Mekelle Citywith the study population comprised of all delivered mothers in selected ketenas^2^ of Mekelle City.

### Sampling technique and procedure

A sample of 564 participants were selected using the single population proportion formula with the following assumptions: proportion (P) of PCC experience which is 38.2% conducted in Ethiopia [12], a confidence level (CI) of 95%, marginal error (d) 5, 5% non-response and considering a design effect of 1.5. A multi-stage cluster sampling technique was employed. In the first stage, three sub-cities were selected randomly and from the fourteen tabias of these selected sub-cities. In the second stage, five tabias of the 14 were chosen and then two ketenas from each of the tabias were select by simple random sampling (SRS) technique.

### Data collection instrument and quality management

Data was collected using a structured pre-tested questionnaire through a face-to-face interview. The questionnaire was informed by reviewed literature emanating from the relevant research area. Prior to data collection, validity and reliability testing of the instrument was undertaken. To examine content validity, a panel of experts was asked to review and refine each item in the instrument. This panel was composed of three senior midwives and/or nurses, one obstetrician, and two researchers in the field of reproductive health. The items had strong internal consistency (α = 0.76) among a sub-sample of participants (*n* = 30). Data quality was assured by trained Bachelor of Science Degree holders who were fluent in the local language; Tigrigna.

Daily supervision, spot checking, and review of the completed questionnaires was conducted by the research assistants.

### Variables

Utilization of PCC was the outcome of interest and the independent variables were demographic characteristics, obstetric and reproductive health factors, knowledge, as well as family and health care system factors.

### Statistical analyses

Data were coded and entered into EPi-Info™ Version 7 software. Descriptive analysis was done. Binary logistic regression and multivariable logistic regression models were performed to obtain the crude and adjusted odds ratios for the outcome variable, using the SPSS 20™- statistical package. Statistical significance was set at *p* < 0.05.

### Ethical consideration

Ethical clearance was obtained from Mekelle University, College of Health Sciences - Ethical Review Board. In addition, an official letter of cooperation was granted by the Tigray Regional Health Bureau and administrative offices of Mekelle City. The purpose of the study was explained to the study participants and written informed consent was obtained. Participation was on a voluntary basis after written consent and confidentiality were secured.

## Results

### Socio-demographic characteristics

A total of 561 mothers who delivered within the last year (12 months or less) were included in the study with a response rate of 99.5%. Of the 561 participants, about half of the mothers (277; 49.4%) were within the age group 25–34 years and ranged from 18 to 43 years. The mean age of the participants was 31.22 years (±6.04).

Majority of study participants (536; 96.1%) were from Tigray ethnic group, and 232(41.4%) were house wives by occupation. More than three-quarters (438; 78.1%) of the participants were married, with over 90% (580) belonged to the Orthodox Christian religion and one third (191; 34%) had secondary school education (Table 1).

**Table 1:** Socio-demographic characteristics of participants in North Ethiopia, 2018 (*N* = 561)

Variable	Frequency	Percent (%)
Age		
15–24	88	15.7
25–34	277	49.4
35–45	196	34.9
Mother’s education		
No formal education	74	13.2
Can read and write	73	13.0
Elementary school	127	22.6
Secondary school	191	34.0
College/university	96	17.1
Marital status		
Married	438	78.1
Single/never married	62	11.1
Divorced	53	9.4
Widowed	8	1.4
Occupation		
Housewife	232	41.4
Daily worker	94	16.8
Government employee	104	18.5
Private business	129	23.0
Student	2	0.4
Religion		
Orthodox	508	90.6
Muslim	42	7.5
Others ^a^	11	2.0
Ethnicity		
Tigray	539	96.1
Amhara	13	2.3
Afar	9	1.6
Income of respondents in ETB		
Less than 500	132	23.5
501–1000	207	36.9
1001–2000	151	26.9
2001 and above	71	12.7
Time spent to reach health facility		
Less than 30 min	322	57.4
30 min and above	239	42.6
Means of transport to reach facility		
Foot	303	54.0
Public transport	187	33.3
Private transport	71	12.7

^a^ Other religions = Catholic and Protestant

### Obstetrics and reproductive health characteristics

Assessment of obstetric and reproductive health characteristics revealed that about 370(67.6%) participants had between two to four pregnancies. Concerning parity, three-quarters (423; 75.4%) of participants were multipara. Six out of ten (341; 60.8%) respondents had a history of family planning use. On the subject of previous adverse birth outcomes, 118(21%) had a history of one or more adverse birth outcomes. Of those, who had adverse birth outcome, 67(56.8%), 22(18.6) and 23(19.5%) of participants reported that they had experienced abortion, still birth, and congenital malformations respectively.

Regarding access to PCC, six out of ten mothers 62(60.8%) received the care from hospital. Thirty-nine (7%) of respondents had a chronic health problem. With respect to husband/partner support on PCC, 159 (28.3%) of participants had a partner support to uptake preconception care and 167(29.8%) had a joint plan discussion with their partner about PCC **(**Table 2).

**Table 2:** Obstetric and Reproductive Health Characteristics, North Ethiopia, 2018 (*N* = 561)

Variable	Frequency	Percent
Gravidity		
1 Pregnancy	113	20.1
2–4 Pregnancies	379	67.6
≥ 5 Pregnancies	69	12.3
Parity		
Primipara (1 delivery)	138	24.6
Multipara (2 & above delivery)	423	75.4
Previous use of family planning		
Yes	341	60.8
No	220	39.2
Previous adverse pregnancy outcome		
Yes	118	21.0
No	443	79.0
Type of adverse pregnancy outcome (*n* = 118) (Multiple responses)		
Congenital anomalies	23	19.5
Low birth weight	17	14.4
Preterm	10	8.5
Abortion	67	56.8
Still birth	22	18.6
Neonatal death	7	5.9
Infection	13	11.0
Place where PCC was received		
Hospital	62	60.8
Health center	24	23.5
Private clinic	16	15.7
Having cultural factor ^a^		
Yes	33	5.9
No	528	94.1
Husband support for PCC		
Yes	159	28.3
No	402	71.7
Joint plan discussion with partner		
Yes	167	29.8
No	394	70.2
Chronic health problem		
Yes	39	7.0
No	522	93.0
Type of chronic health problem(*n* = 39) (Multiple responses)		
HIV/AIDS	14	35.9
HTN	14	35.9
Diabetes mellitus	5	12.8
Others ^b^	8	20.5

^a^ Other chronic health problem: epilepsy, tuberculosis and anemia

^b^ Cultural factor: use traditional medicine, holly water and autonomy deficit to decide

### Utilization of preconception care

**Fig. 1:**
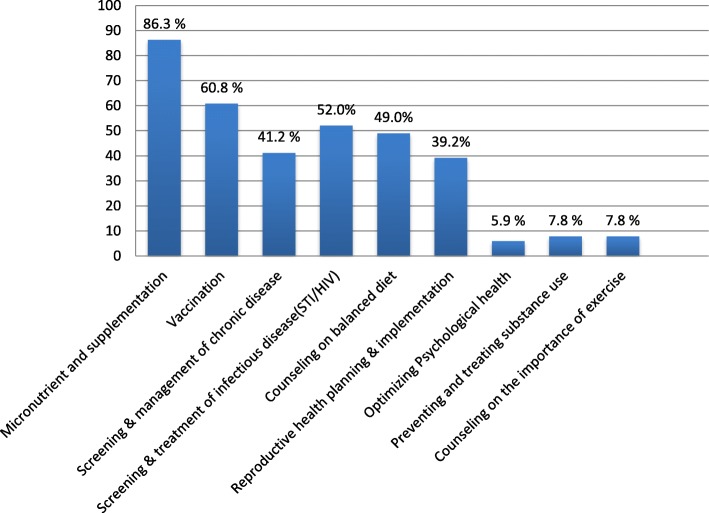
Proportion of World Health Organization components of preconception care utilization among women who delivered within the last one year in Mekelle city, North Ethiopia, 2018 (*N* = 561)

Among the 561 participants, 102(18.2%) mothers had utilized at least one component of the World Health Organization package of PCC services before their last baby. The most commonly utilized component of PCC in this study was micronutrient supplementation (i.e., iron, folic acid) 88(86.3%) whereas the least utilized was optimizing psychological health (5.9%) (Fig. 1).

### Predictors for the utilization of preconception care

In this study, results of the bivariable logistic regression showed that mothers’ age, level of education, monthly income, history of family planning use, experience of adverse birth outcome, joint discussion plan with partner, chronic health problem, challenge to visit heath facility, husband support and knowledge on preconception care were associated with utilization of PCC.

Through multivariable logistic regression, a history of adverse birth outcome, chronic health problem, husband support, challenge to access health facility, and knowledge on preconception care was found to have significant statistical association with utilization of PCC.

Mothers who were knowledgeable about PCC had two times higher odds of service utilization when compared to their counter parts (AOR: 2.21; 95% CI: 1.03, 4.73).

The odds of PCC utilization were five times higher among mothers who had a history of adverse pregnancy outcomes compared those who did not experience an adverse event (AOR:5.1; 95% CI:2.31,11.24).

In addition, mothers who had chronic health problems were nearly six times more likely to utilize PCC compared to their non-chronic disease afflicted counterparts (AOR: 5.69; 95% CI: 2.06, 15.72). This study revealed that husband/partner support has a significant effect on the PCC service utilization. Mothers who received support from their husbands were nearly 14 times more likely to utilize PCC than participants who did not have the support of their husband (AOR: 13.84;95% CI: 6.02, 31.79). However, mothers who had any challenges in visiting health facilities were 76% less likely to utilize PCC than mothers who had no such challenge (AOR: 0.24; 95% CI: 0.16, 0.48) (Table 3).

**Table 3:** Association between different variables and preconception care among women who delivered within the last one year in Mekelle city, North Ethiopia, 2018 (*N* = 561)

Variable	Preconception Care Utilization		Odds Ratio and 95% CI	
	Utilized (%)	Not utilized (%)	Crude	Adjusted
Age (years)				
15–24	22(25.0%)	66(75.0%)	1	1
25–34	58(20.9%)	219(79.1%)	0.79(0.45–1.39)	1.23(0.50–3.04)
35–45	22(11.2%)	174(88.8%)	0.37(0.19–0.73)*	1.34(0.42–4.24)
Mother’s education				
No education	26(17.7%)	121(82.3%)	1	1
Primary education	11(8.7%)	116(91.3%)	0.44(0.21–0.93)*	0.59(0.20–1.70)
Secondary education	37(19.4%)	154(80.6%)	1.12(1.01–1.95)*	1.26(0.51–3.13)
More than secondary education	28(29.2%)	68(70.8%)	1.92(1.04–3.53)*	1.24(0.45–3.43)
Household income (ETB)				
Less than 500	36(27.3%)	96(72.7%)	1	1
501–1000	25(12.1%)	182(87.9%)	0.37(0.21–0.65)**	0.46(0.19–1.06)
1001–2000	25(16.6%)	126(83.4%)	0.53(0.29–0.94)*	0.61(0.25–1.52)
Above 2000	16(22.5%)	55(77.5%)	0.78(0.4–1.53)	0.70(0.25–1.94)
Previous family planning use				
No	25(11.4%)	195(88.6%)	1	1
Yes	77(22.6%)	264(77.4%)	2.28(1.39–3.71)**	1.55(0.74–3.23)
Previous adverse birth outcome				
No	62(14.0%)	381(86.0%)	1	1
Yes	40(33.9%)	78(66.1%)	3.15(1.98–5.02)**	5.10(2.31–11.24)**
Joint plan discussion with partner on preconception care				
No	23(5.8%)	371(94.2%)	1	1
Yes	79(47.3%)	88(52.7%)	14.48(8.62–24.34)**	2.05(0.88–4.78)
Having any chronic health problem				
No	77(14.8%)	445(85.2%)	1	1
Yes	25(64.1%)	14(35.9%)	10.32(5.14–20.73)**	5.69(2.06–15.72)*
Having any challenge to access health facility				
No	82(35.5%)	149(64.5%)	1	1
Yes	20(6.1%)	310(93.9%)	0.11(0.07–0.2)**	0.24(0.16–0.48)**
Having husband/partner support				
No	17(4.2%)	385(95.8%)	1	1
Yes	85(53.5%)	74(46.5%)	26.0(14.61–46.33)**	13.84(6.02–31.79)**
Knowledge index on PCC				
Not knowledgeable	56(11.6%)	428(88.4%)	1	1
Knowledgeable	46(59.7%)	31(40.3%)	11.34(6.65–19.34)**	2.21(1.03–4.73)**

*P-value < 0.05; **P-Value < 0.001

## Discussion

Understanding of PCC is critical for many Sub-Saharan African countries, such as Ethiopia, where maternal and perinatal mortality remains alarmingly high. This community based cross-sectional study identified factors influencing PCC utilization among mothers who gave birth within the last one year in Mekelle City, north Ethiopia.

The findings of this study showed that 102(18.2%) of the mothers had utilized PCCS services. This is lower than previous findings in Ethiopia [12], China [13], and Los Angeles [14]. This discrepancy might be due to the difference in access to information, socioeconomic status, and the quality of health care delivery system. However, PCC utilization is higher than a study conducted in Debremarkos town, Ethiopia where only 8.4% of the pregnant mothers reported using PCC [15]. This variability may be related to differences in the study population’s level of education, culture, and the study setting.

Regarding the predictors, this study revealed that knowledge of mothers were associated with PCC utilization, Which was in line with the findings reported from Debremarkos, Ethiopia [15], Oromia, Ethiopia [12], China [13], and Philadelphia [16]. This might relate to information leading to knowing details about the service rather obligations to utilize the service. This suggests that it is evident that knowledge was related to the level of practices or utilization of the services.

According to the current study, husband/partner support has a significant effect on the service utilization of PCC. This finding is consistent with studies done in Ethiopia (Debremarkos) [15], Ethiopia (Oromia) [12] and China [13] which found that the participants who had support from their husbands towards PCC showed increased compliance. This may be due to having reproductive health policies including male engagement strategies promoting maternal and child health care service.

Mothers who had a history of adverse birth outcomes were positively associated with PCC utilization, which was also seen in a study done in Los Angeles [14] indicating previous adverse infant outcomes being associated with increased odds of having utilized PCC in the most recent pregnancy. Potentially mothers’ who had an experience of adverse birth outcomes were more conscious and PCC compliant in their subsequent pregnancies.

Finally, this study noted that PCC utilization was negatively associated with mothers who had challenges to access health facilities. Similarly, studies from China [13], and Sudan [17] showed that having challenges to access/visit health facilities was positively associated with mothers’ poor service utilization. Challenges such as lack of finances, unplanned pregnancies, and poor quality of care services are potential contributory barriers to mothers’ lack of uptake of PCC services.

## Conclusions

This study found that PCC is low in the select Ethiopian population. Factors which increased utilization of PCC include more health literate mothers, prior adverse birth outcomes, pre-existing chronic health conditions, and husband/partner support. Conversely, barriers to access health care facilities were found to contribute to reduced utilization of PCC.

These findings may inform future scale up of PCC and strengthen PCC units to meet the pressing needs of this population. Consideration of barrier-free (i.e., cost reduced) PCC services and development (or adoption) of evidence-based guidelines to enhance PCC may catalyze such efforts. Further inclusion of male partners needs to be promoted.

It is recommended that mixed methods research, program evaluations, and longitudinal research efforts be undertaken to explore and address the imperative for PCC.

## Data Availability

The datasets used and/or analyzed during the current study is available from the corresponding author on request.

## Abbreviation

PCC: Preconception care services

## Footnotes

1 Tabia: The unit of administration consists of 3 and above ketena or 10–15,000 population.

2 Ketena: The smallest unit of administration consists of a minimum of 4000 population, but sometimes depends on geographical location.
